# Supplementary figures and images for: Manual dexterity of mice during food-handling involves the thumb and a set of fast basic movements
Source: PLoS One. 2020 Jan 15;15(1):e0226774. doi: 10.1371/journal.pone.0226774 (PMC6961851; doi:10.1371/journal.pone.0226774)

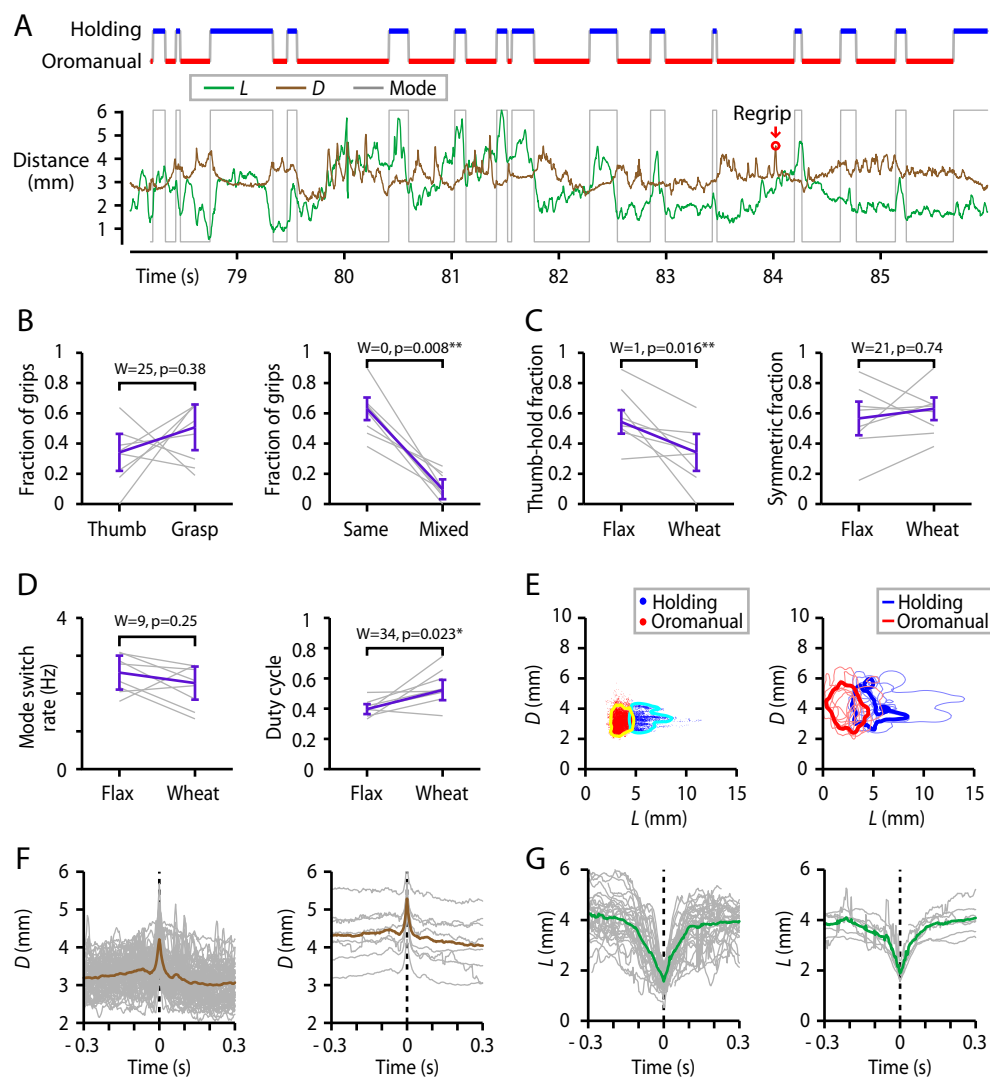

Suppl. Fig. S1

Supplement: S1 Fig — (A) Top: Example oromanual/holding ethogram for a mouse feeding on a wheat berry. Bottom: Time series of L, the distance between the nose and the average D3 position (green), and D, the distance between the D3 digits (brown). The same ethogram is also shown (gray). (B) Left: Relative frequencies of grip types; indeterminate hold types are not included. Gray lines: individual mice (n = 8), error bars: median ± m.a.d. across mice. The lower rate of thumb-holds was the main difference compared to flaxseed (see text). Right: Relative frequency of symmetric (i.e., same on both hands) and asymmetric grip types as a fraction of all grip types. (Not shown: indeterminate grip types.) (C) Fraction of thumb-holds (left) and symmetric grips (right) for flaxseed versus wheat berries. (D) Mode switch rate (left) and duty cycle (right) for flaxseed versus wheat berries. (E) Left: Example of k-means clustering (k = 2) of L and D, for the video shown in (A). Blue dots correspond to the holding phase and red the oromanual phase. Cyan and yellow lines show the 10% contour line of 2D kernel smoothed density of the points in the respective clusters. Right: L-D clustering for all mice. Light blue and red lines show the 10% maximum density contours lines for the holding and oromanual clusters, respectively, for each of n = 8 mice. Dark blue and red show the 10% contour lines of the average kernel smoothed density across all mice. (F) Left: Peak-aligned individual (gray) and average (brown) regripping events for all wheat-handling videos from one mouse. Right: Average regrip traces from each mouse (gray, n = 8) and the overall average (brown). (G) Left: Peak-aligned individual (gray) and average (green) sniffing events for all wheat-handling videos from one mouse. Right: Average sniff traces from each mouse (gray, n = 6 mice) and the overall average (green). (PDF) [file pone.0226774.s001.pdf]

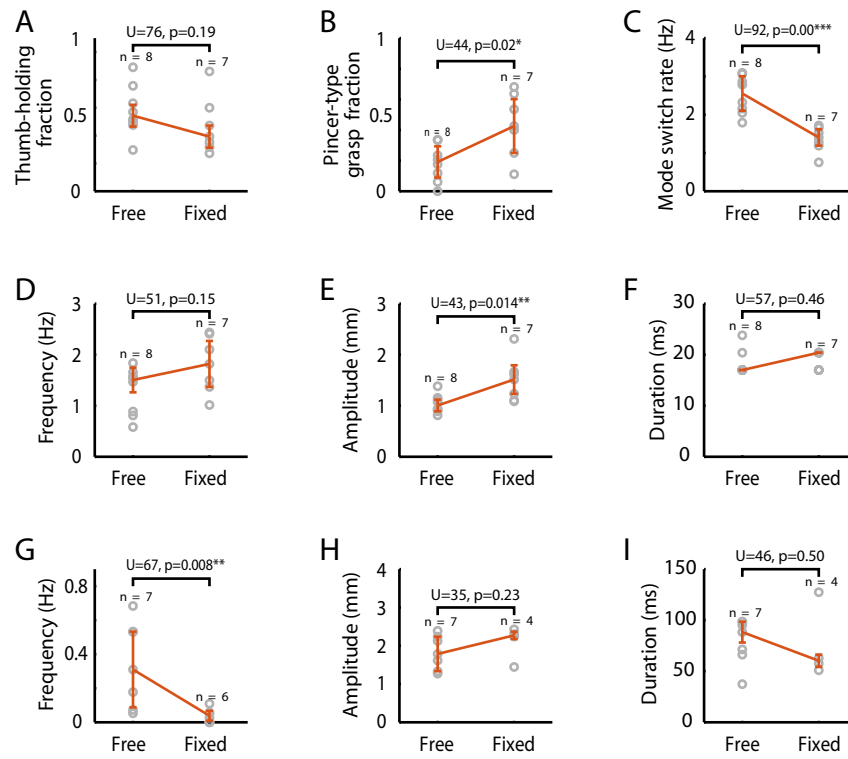

Suppl. Fig. S2

Supplement: S2 Fig — Gray circles: individual mice; error bars: median ± m.a.d. (A) Fraction of thumb-hold grips. (B) Fraction of pincer-type grasps. (C) Mode switch rate. (D) Regrip frequency. (E) Regrip amplitude. (F) Regrip duration. (G) Sniff frequency. Mice with insufficient ventral view data excluded. (H) Sniff amplitude. Mice with no detected sniffs excluded. (I) Sniff duration. Mice with no detected sniffs excluded. (PDF) [file pone.0226774.s002.pdf]

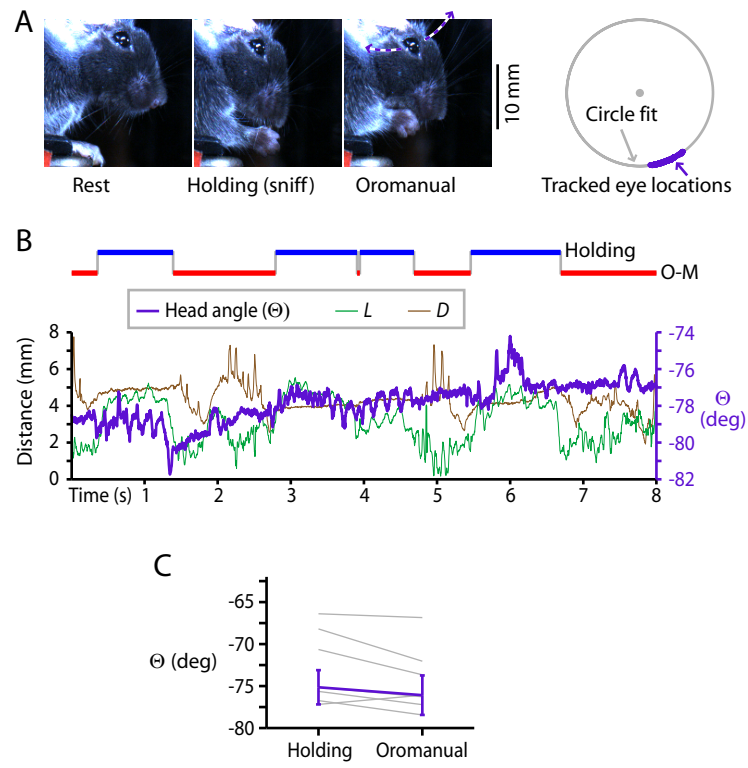

Suppl.Fig. S3

Supplement: S3 Fig — (A) Left: Side-view images of a head-fixed mouse at rest (left) and during the holding (middle) and oromanual (right) phases. Purple dashed line shows the arc swept by the eye as the head rotates about the head-fixation rod. Right: Measurement of head angle (Θ). An example is shown of the individual eye locations measured in the video (purple dots), with the center and circumference (gray) of the circle that best fits these points according to Pratt’s method (see Methods). (B) Top: Example ethogram of handling behavior for a partially head-fixed mouse feeding on a flaxseed, based on manual annotation of holding (blue) versus oromanual (red) phases. Bottom: Time series of the tracked L3D, the three-dimensional distance between the nose and the average D3 position (green trace); D, the distance between the D3 digits (brown trace); and Θ the angle of the eye about the center of rotation (purple trace). (C) Head angle Θ during holding versus oromanual phases for partial head-fixation. Thin lines are individual mice, error bars are median ± m.a.d over all mice (n = 7). (PDF) [file pone.0226774.s003.pdf]
